# How to use curriculum mapping to ensure a coherent and coordinated learning spiral in a competency-based medical curriculum across two medical universities

**DOI:** 10.1186/s12909-025-07837-w

**Published:** 2025-10-07

**Authors:** Tuija Eeva Elisabeth Waldvogel, Monika Link, Giovanni Pedrazzini, Jacqueline Jennebach, Jörg Goldhahn, Olaf Ahlers

**Affiliations:** 1 Department of Health Science and Technologies, ETH Zurich, Zurich, Switzerland; 2 Faculty of Biomedical Sciences, USI, Università della Svizzera italiana, Lugano, Switzerland; 3 German Association of Medical Faculties, Berlin, Germany; 4 Brandenburg Medical School Theodor Fontane, Institute of Research in Health Sciences Education, Faculty of Health Sciences Brandenburg, Neuruppin, Germany; 5 Institute of Medical Informatics, LOOOP Project, Charité – Universitätsmedizin Berlin, Berlin, Germany

**Keywords:** Curriculum mapping, Curriculum evaluation, Learning spiral, Competency-based medical education

## Abstract

**Background:**

Evaluation of competency-based medical curricula is still a challenge. Curriculum mapping comprises all learning objectives for the learning events which are (usually) mapped to a national framework. This study evaluates coherence within the learning spiral across two consecutive competency-based curricula by usage of curricular maps.

**Methods:**

Curriculum mapping data of two undergraduate medical curricula (Bachelor and consecutive Master) from two different Swiss universities was used to evaluate a given topic (in our case cardiology) related to continuity and increasing complexity. In addition, coverage of the Swiss national framework (’PROFILES’) was assessed.

**Results:**

A continuous exposure to cardiovascular content across the two programs as well as an increasing complexity was found. The analysis further showed that most parts of the national Swiss framework (‘PROFILES’) are covered to some extent and revealed missing coverage of some parts of the first chapter (‘General Objectives’) and second chapter (‘Entrustable Professional activities’).

**Conclusion:**

The results support the implicit notion that the medical curriculum across two universities can be coherent and provide the necessary structure to enable a coordinated learning spiral. The approach can be used for any curriculum which has been mapped to a framework to evaluate the coherence and coordination of a learning spiral in each field. This approach can be very valuable especially for medical programs where students change from one institution to another.

**Supplementary Information:**

The online version contains supplementary material available at 10.1186/s12909-025-07837-w.

## Background

During the last decades, various debates focused on the use of competency-based medical education (CBME) resulting in wide adoption of the concept of medical curricula worldwide [1]. CBME focuses on the attainment of competencies, and it is thus considered an outcomes-based approach [2, 3]. It is used to design, implement, assess, and evaluate medical education programs [4, 5]. The use of national or international competency frameworks is a common approach to embrace CBME and to prepare students for practice [1].

Curriculum mapping (CM) is utilized to demonstrate how pre-defined outcomes of a competency-based framework are addressed within a medical program [4, 6–8]. CM provides the possibility to review, evaluate and revise a curriculum, which are key attributes when designing a medical curriculum [9]. A transparent and easily accessible curriculum map enables users to view the links between curriculum elements [10] whereby ‘viewing the learning spiral by filtering the progression in terms of breadth, depth, utility and proficiency, as reflected in learning objectives […]’ is made possible ([11], p.27). There is increasing literature on the experiences with different mapping approaches [4, 12–14] and some methodological work, where Watson and colleagues proposed a typology for curricular mapping for health professions education [15]. Yet there is little literature on the specific use of curricular maps for the evaluation of the alignment and learning spiral of a curriculum [1].

### Situation in Switzerland

Medical faculties in Switzerland had to implement new competency-based medical curricula at different universities within a system of Bachelor’s and Master’s degrees. This was the consequence of the mandatory national Swiss outcome catalogue for medical education ‘Principal Relevant Objectives and Framework for Integrated Learning and Education in Switzerland’ (PROFILES) [16, 17]. PROFILES define the content and aims of medical training in Switzerland and are composed of three chapters: General Objectives (GO), Entrustable Professional activities (EPA) and Situations as Starting Points (SSP). The GO chapter is based on the CanMEDS 2015 Framework which was originally developed by the Royal College of Physicians and Surgeons of Canada [17, 18]. The EPA chapter depicts the main medical tasks that a physician must be able to perform autonomously on the first day of residency [16, 19]. The SSP chapter lists 265 situations which cover the common symptoms, complaints and findings that a resident should be able to manage with respect to all age groups and in any type of setting [16, 20]. PROFILES are the basis for the national Federal Licensing Examination (FLE) which every graduate must pass to begin postgraduate training [16, 20].

After implementation of PROFILES in 2017 the faculties of medicine decided to establish a Swiss working group and defined several key factors for successful implementation of PROFILES. CM was identified as one of the key factors to ensure that the planned curriculum covers all the elements of PROFILES over a 6-year program at the medical faculties [20].

Based on PROFILES, the medical faculties in Switzerland had to create or adapt their curriculum appropriately [20]. In Switzerland, the universities have high autonomy concerning the structure of their undergraduate medical curriculum since the implementation of the new Federal Law concerning university medical professions in 2007 [21]. Several new Bachelor and Master programs were launched starting in 2017. In some cases, students must complete their studies with a Master’s degree at another institution after the Bachelor. Thus, it was crucial to create a coherent competency-based curriculum including an effective learning spiral from the beginning of the Bachelor till the end of the Master.

### Curriculum development and evaluation

Development of competency-based medical curricula is well documented by Patricia Thomas and colleagues, who described six steps ending with the evaluation of the program in order to re-adapt the curriculum [22]. The evaluation of a competency-based medical curriculum is still a major challenge [7].

With this study we contribute to overcoming this challenge with an analysis of a longitudinal curriculum taught at two Swiss universities: Students study the first three years (Bachelor of Medicine) at Swiss Federal Institute of Technology (ETH) and 50% of these students finish their studies with their Master of Medicine (three years) at Università della Svizzera italiana (USI). Both universities designed their respective program from scratch based on the PROFILES within the ‘Learning Opportunities, Objectives and Outcomes Platform’ (LOOOP) [23–25]. This platform is used by the international LOOOP network for Research in Health Sciences Education for about 250 programs from 51 countries [25]. It is an online curriculum mapping tool, where the content of a medical program can be displayed. For each module, respective learning events (LE) consist of one or more learning objectives (LO) which can be linked (‘mapped’) to different catalogues (such as PROFILES). The curricular map of ETH-Bachelor was used as a basis for the development of the curriculum of the USI-Master.

Both institutions have an organ system based integrative curriculum that embraces CBME. An integrated curriculum is defined as “a fully synchronous, transdisciplinary delivery of information between the foundational sciences and the applied sciences throughout all years of a medical school curriculum.” according to the AMEE Guide No. 96 [26]. More specifically it is the combination of a horizontal and vertical integration, the former being the integration across disciplines within a finite period of time and the latter the adoption of a Z-shaped curriculum model where biomedical sciences are combined with clinical cases from the very beginning. This combination of horizontal and vertical integration is the most ideal form of integration and can also be named as ‘spiral integration’. Based on this spiral integration, the concept of the ‘learning spiral’ refers to a curriculum where students revisit topics with increasing complexity over time to reach a certain competency [26, 27]. The coherence and coordination of integrated curricula is deemed crucial by the US accreditation institution, Liaison Committee on Medical Education [28]. In addition, based on the curricular structure and the presence of a comprehensive curricular map at both institutions, this study was designed to evaluate whether the creation of the mentioned two undergraduate medical programs was successful in providing a meaningful ‘learning spiral’. We are not aware of any scientific publication that describes such an analysis of one common curriculum represented by two curricular maps across two universities.

## Methods

### Aim of the study

In this study, cardiology-related content of ETH-Bachelor and USI-Master was analysed to assess the continuity, the increase in complexity over time and the coverage of the national framework (PROFILES). Cardiology-related content was chosen as an example because it is represented in several modules over six years and is present as an organ system at both institutions.

Therefore, the following hypotheses and research question were formulated:

1. The cardiovascular content is longitudinally distributed over six years (‘*continuity’*).
2. The mapping depth of the cardiovascular content in the Master is higher than in the Bachelor (‘*external complexity*’).
3. The mapping depth within the Bachelor and within the Master respectively increases over the study years (*‘internal complexity’*).
4. How are GO and EPA covered by the cardiology curricula?

To answer the hypotheses and research question, we used data from two curricular maps of the ETH-Bachelor and the USI-Master, which were scanned for cardiovascular content. For this analysis the number of mappings was used for calculations. Higher numbers of mappings indicate higher coverage of the respective learning objective (LO).

### Specification of used data

Curricular maps of ETH-Bachelor and USI-Master are characterized as follows: Each LO belongs to one of the following domains: knowledge, skills, or attitudes. Each LO is mapped to at least one PROFILES item, yet multiple PROFILES can be mapped per LO.

LO were formulated by the lecturer of each LE when setting up the LE during curriculum development. CM specialists from each institution collected the LO and conducted a quality control of these LO. The mapping, which is defined as a combination of a LO with at least one PROFILES item, was done by the CM specialists and reviewed by the respective lecturers. The curricular maps were created in close collaboration between the CM specialists of the institutions based on a common mapping procedure. The following established mapping criteria were used:

- For each LO all three PROFILES chapters (GO, EPA, SSP) can be mapped (multiple PROFILES items per LO possible).
- EPAs were also mapped if the skill was not demonstrated, but only addressed theoretically.
- PROFILES items were only mapped to LO, if the central aspect of the PROFILES item was fulfilled by the LO.

Based the manual mapping of LO with PROFILES by the CM specialist, the LOOOP database automatically assigned a mapping depth according to LOOOP’s Taxonomy [25]. LOOOP’s Taxonomy is an adapted combination of Miller’s Pyramid [29] and the revised Bloom’s Taxonomy [30]. Based on the level of the verb used, it reflects the complexity of the curricular content (see Table 1). Mapping depth was used to evaluate the complexity of the curriculum along the six years.

**Table 1:** Mapping depths used in LOOOP’s Taxonomy for the declaration of the complexity of the mappings and corresponding levels of modified Bloom’s Taxonomy and Miller’s Pyramid

Mapping depth	Description	Bloom’s taxonomy (knowledge)	Miller’s pyramid
1	Factual knowledge: knowledge of terminology, specific details and elements	Remember	Knows
2	Conceptual knowledge: knowledge of principles and generalizations, theories, models and processes	Understand Apply Analyse Evaluate	Knows how
3a	Professional activities: ability to show in simulation	Create (in simulation)	Shows (skills, attitudes)
3b	Professional activities: ability to act in practice	Create (in practice)	Does (skills, attitudes)

### Definition of the dataset

Data from the academic year 2023/2024 was used for the analysis. The three years of ETH-Bachelor were completely mapped, while no LO were mapped for the ninth semester within the last year of USI-Master, as it corresponds to clinical activities conducted in different clinical departments and is thus different for each student.

To identify cardiovascular content within the mentioned eleven semesters, the following information was extracted from LOOOP: semester, module, LE title, LO, PROFILES and mapping depth. The final dataset was created in three steps: 1. identification of mappings and export, 2. merging of data and removal of duplicates, and 3. final plausibility check.

#### Step 1: identification of mappings and export from LOOOP

Cardiology-related mappings were identified in LOOOP based on four approaches for ETH-Bachelor and USI-Master, respectively. The aim of using four approaches was to identify as much cardiology-related content as possible.

- Approach A: Identifying all mappings to SSP (PROFILES) with cardiology related symptoms.
- Approach B: All mappings within the cardiology modules.
- Approach C: All mappings of learning events related to the discipline ‘cardiology’.
- Approach D: All mappings of LO, which were identified by a cardiology related text search (search terms: ‘heart’, ‘cardio’, ‘circulation’, ‘blood pressure’).

All identified mappings were exported as Excel documents. A detailed list of used PROFILES (approach A) and modules (approach B) can be found in the additional data 1.

#### Step 2: merging of data and removal of duplicates

The extracted Excel documents were merged into one Excel document. Duplicates were identified by combining the text of the LO with the text of the mapped PROFILES using the ‘textjoin’ function in Excel. Afterwards, all duplicates were removed.

#### Step 3: plausibility check

A final plausibility check was conducted to exclude mappings of LO that don’t concern cardiology. These mappings were removed.

### Data analysis

The final data set was analysed and visualized with Microsoft Excel (Version 2411) and SPSS (Version 29.0.1.0). Significant differences were assumed by *p* < 0.05.

1. *Continuity*
  Continuity was evaluated by descriptive analysis of the LO and mappings for ETH-Bachelor and USI-Master.
2. *External Complexity*
  Evolution of the complexity from ETH-Bachelor to USI-Master was analysed by graphs. Differences between both programs were assessed by Mann-Whitney U test.
3. *Internal Complexity*
  Complexity within ETH-Bachelor and within USI-Master was also analysed by graphs. Differences within each program were assessed by Kruskal-Wallis-test followed by pairwise comparison with Bonferroni correction for repeated measurements.
4. *Coverage of PROFILES*

A detailed descriptive analysis of the number of mappings in each GO subchapter (see Table 2) and each EPA subchapter (see Table 3) was conducted for ETH-Bachelor and USI-Master. The aim was to show the coverage of the respective aspects within the cardiology curriculum. The SSP subchapter was not scrutinized in detail because the number of mappings would be biased due to SSP usage for dataset selection within approach A.

**Table 2:** Explanation of general objectives (GO) within PROFILES [17]

GO 1 Medical Expert	Synthesizes the key aspects of undergraduate training, including medical knowledge and skills, such as taking a medical history, performing a triage, physical examination, clinical reasoning, communication with patients, include ethical, critical awareness of economic, ethical, and societal aspects
GO 2 Communicator	stablish and maintain effective relationships with patients and relatives
GO 3 Collaborator	Communication and collaboration skills for the work in interprofessional and interdisciplinary teams
GO 4 Leader/Manager	Leadership skills concerning topics such as insurance, economy, public health and pandemics through their activities as clinicians, administrators, scholars, or teachers
GO 5 Health Advocate	Promotion of the importance of public health and preventive healthcare for the individual patient, for patient groups, and for society
GO 6 Scholar	Recognizing the need for lifelong learning and continual updating of their professional expertise
GO 7 Professional	comprises ethical and personal standards, maintenance of the physician’s own health and the accountability to the profession and society

**Table 3:** Titles of entrustable professional activities (EPA) within PROFILES [17]

EPA 1	Take a medical history
EPA 2	Assess the physical and mental status of the patient
EPA 3	Prioritize a differential diagnosis following a clinical encounter
EPA 4	Recommend and interpret diagnostic and screening tests in common situations
EPA 5	Perform general procedures
EPA 6	Recognize a patient requiring urgent / emergency care, initiate evaluation and management
EPA 7	Develop a management plan, discuss orders and prescriptions in common situations
EPA 8	Document and present patient’s clinical encounter; perform handover
EPA 9	Contribute to a culture of safety and improvement

## Results

### Continuity

Number of mappings, LO and the average mappings per LO are displayed in Table 4. Overall, there were more mappings and LO in the ETH-Bachelor while the average number of mappings per LO was higher in the USI-Master.

**Table 4:** Total number of PROFILES mappings, learning objectives and mappings per learning objective for cardiology-related content in ETH-Bachelor and USI-Master per study year

	Mappings	LO	mean mapping/LO	min mapping/LO	max mapping/LO
ETH-Bachelor	1798	583	3	1	15
year 1	858	291	3	1	15
year 2	390	107	4	1	17
year 3	550	185	3	1	10
USI-Master	1346	241	6	1	26
year 4	1149	168	7	1	26
year 5	107	55	2	1	6
year 6	90	18	5	1	8
Grand total	3144	824	4	1	26

*LO* Learning objective, *mapping* number of linkages of an LO with a PROFILES item, *mapping/LO * number of mappings per LO

#### Continuity within ETH-bachelor

**Fig. 1:**
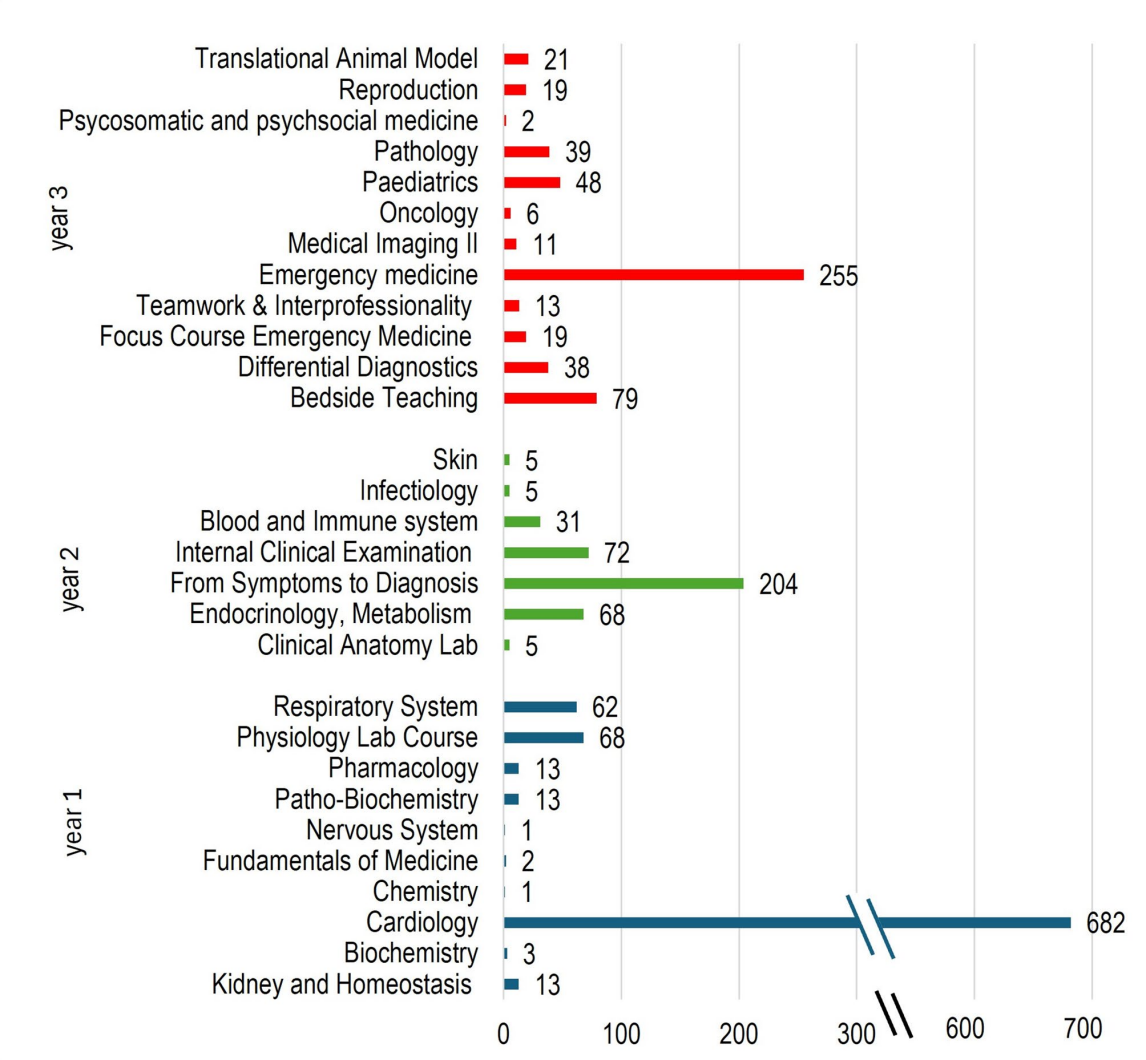
Number of mappings for the cardiology curriculum within ETH-Bachelor displayed by different years and their respective modules

Distribution of the cardiology-related mappings within ETH-Bachelor (Fig. 1) showed a clear peak for the module ‘Cardiology’ in year 1. In the second year, distribution of the mappings revealed a moderate peak for the module ‘From Symptom to Diagnosis’. In the third year the distribution of the mappings revealed a peak for the ‘Emergency Medicine’ module. Overall, the cardiovascular content was mapped in 29 different modules within ETH-Bachelor resulting in 1798 mappings in total. The full study program of ETH-Bachelor can be found in the additional data 2.

#### Continuity within USI-master

**Fig. 2:**
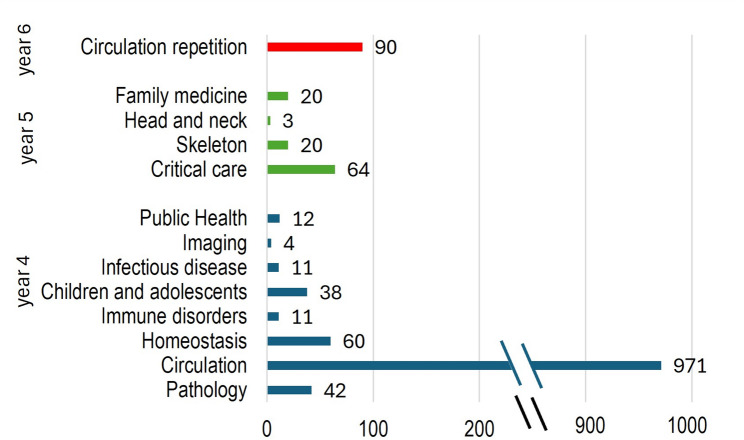
Number of mappings for the cardiology curriculum within USI-Master displayed by different years and their respective modules

Distribution of the cardiovascular mappings within USI-Master (Fig. 2) revealed a clear peak for the cardiology module ‘Circulation’ in year 4. In the fifth year, distribution of the mappings to four modules revealed a moderate peak for the ‘Critical care’ module. In the sixth year, there were only mappings in the ‘Circulation repetition’ module. Overall, the cardiovascular content is mapped in 13 different modules within USI-Master with 1346 mappings in total. The full study program of USI-Master can be found in the additional data 2.

### External complexity

**Fig. 3:**
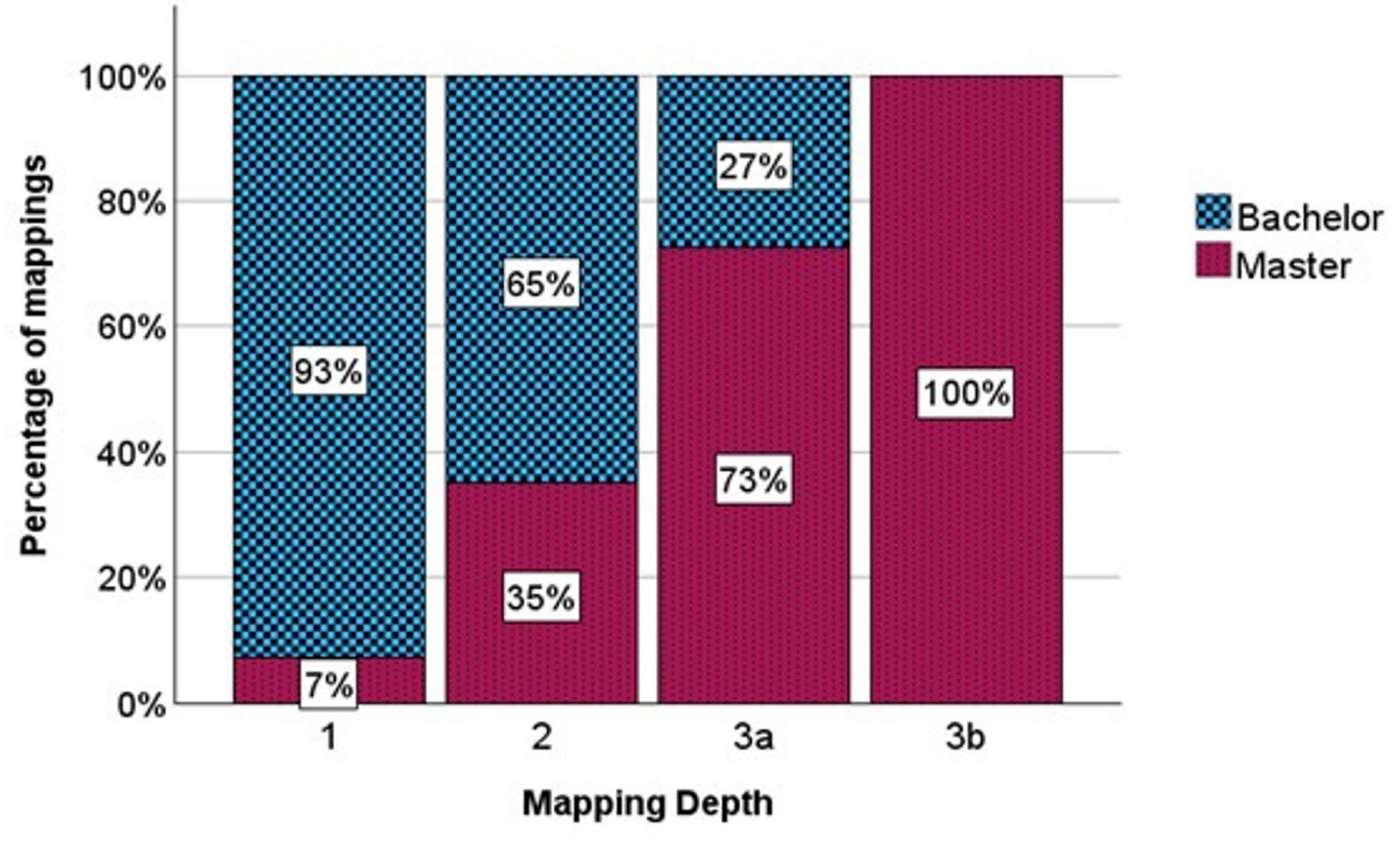
Percentages of mappings for each mapping depth and institution. ETH-Bachelor: blue, USI-Master: red

Graphical analysis of relative distribution of mapping depths between ETH-Bachelor and USI-Master is shown in Fig. 3.

Number of mappings for lower depths was higher for ETH-Bachelor with *n* = 456 at level 1 and *n* = 1175 at level 2 compared to USI-Master with *n* = 36 at level 1 and *n* = 637 at level 2. Number of mappings at level 3a was much higher in USI-Master with *n* = 443 compared to *n* = 16 in ETH-Bachelor. Mappings on level 3b were only found in USI-Master (*n* = 230).

Mann-Whitney U test revealed significant differences between ETH-Bachelor and USI-Master (*p* < 0.001).

### Internal complexity

#### Internal complexity within ETH-bachelor

**Fig. 4:**
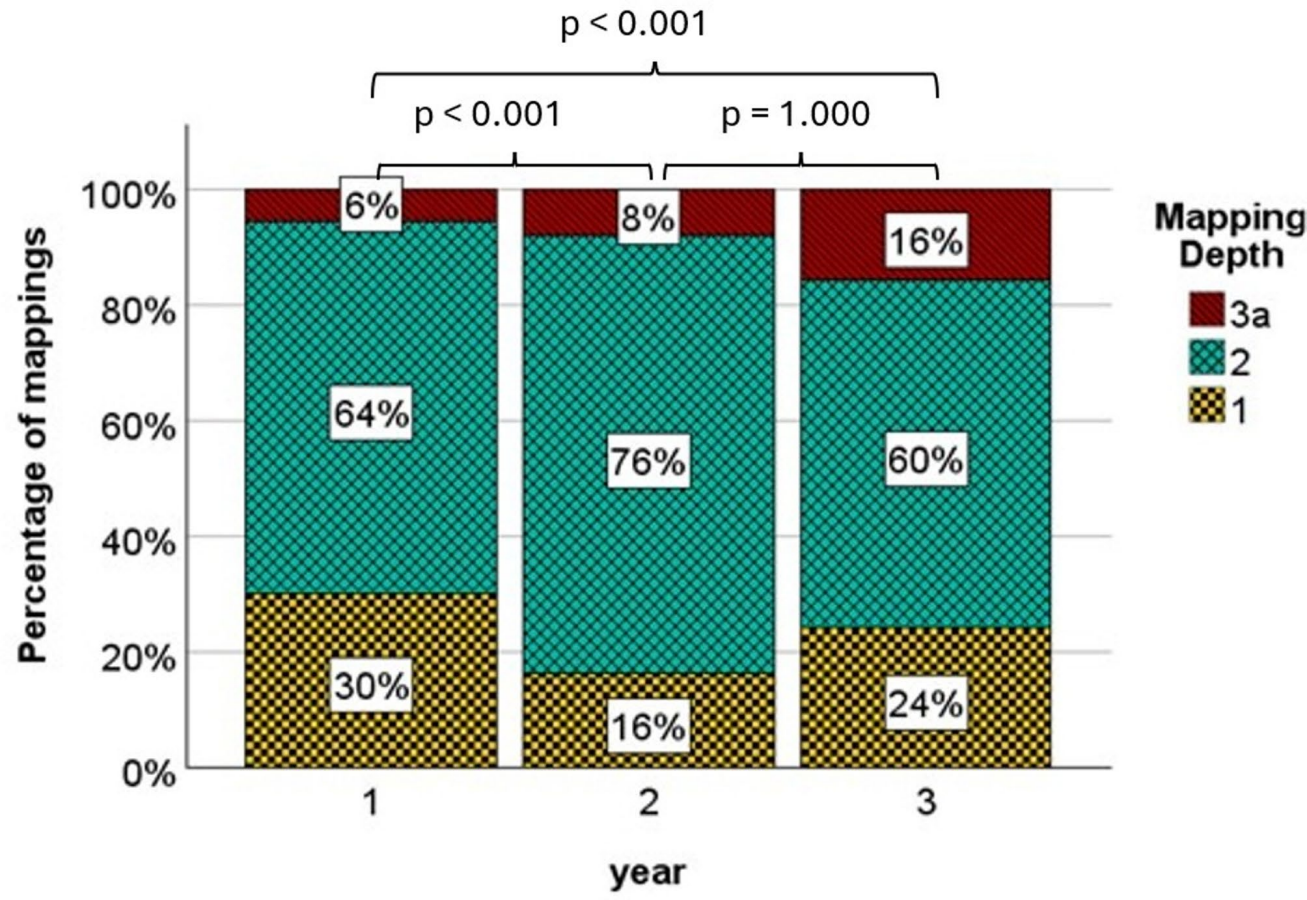
Percentage of mappings for mapping depth 1, 2, and 3a of year 1 to-3 within ETH- Bachelor. *P*-values represent pairwise comparison of mapping depths

Year 1, 2 and 3 contained no mappings on level 3b. The percentage of the mapping depth levels is depicted in Fig. 4 and gives an overview of the distribution of the mapping depths in a study year. Concerning total numbers year 1 had *n* = 259 mappings on level 1, *n* = 55 on level 2 and *n* = 48 on level 3a. Total numbers in year 2 were *n* = 63 on level 1, *n* = 295 on level 2 and *n* = 32 on level 3a. In year 3 total numbers of mapping depth was *n* = 134 on level 1, *n* = 329 on level 2 and *n* = 87 on level 3a.

Kruskal-Wallis-Test revealed significant differences between the study years (*p* < 0.001). Relative complexity within ETH-Bachelor and results of pairwise post-hoc comparison are displayed in Fig. 4.

#### Internal complexity within USI-master

**Fig. 5:**
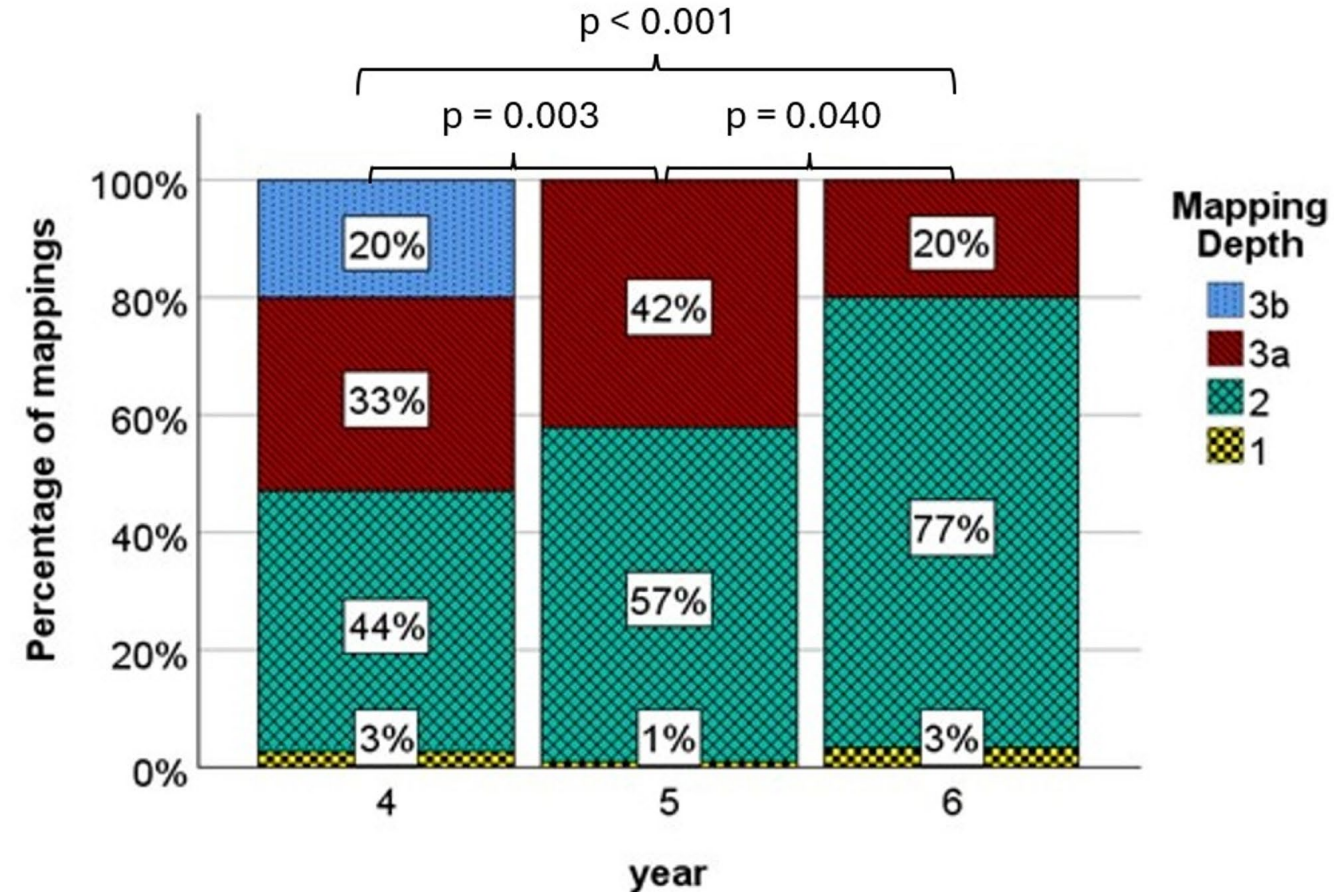
Percentage of mappings for mapping depth 1, 2, 3a, and 3a of year 4 to-6 within USI-Master. *P*-values represent pairwise comparison of mapping depths

The percentage of the mapping depth levels is depicted in Fig. 5 and gives an overview of the distribution of the mapping depths in a study year.

Concerning total numbers year 4 had *n* = 32 mappings on level 1, *n* = 507 on level 2, *n* = 380 on level 3a and *n* = 230 on level 3b. Total numbers in year 5 were *n* = 1 on level 1, *n* = 61 on level 2 and *n* = 45 on level 3a. In year 6 total numbers of mapping depth was *n* = 3 on level 1, *n* = 69 on level 2 and *n* = 18 on level 3a.

Kruskal-Wallis-Test revealed significant differences between the study years (*p* < 0.001). Relative complexity within USI-Master and results of pairwise post-hoc comparison are displayed in Fig. 5.

### Coverage of PROFILES

Numbers and respective percentages of mappings for ETH-Bachelor and USI-Master are listed in Table 5 for GO chapter and Table 6 for EPA chapter.

**Table 5:** Number of mappings per general objective (GO) chapter in total numbers as well as percentages of all GO mappings within ETH- bachelor and USI-Master

	ETH-Bachelor	USI-Master
GO 1 Medical Expert	639 (89.7%)	171 (65.5%)
GO 2 Communicator	5 (0.7%)	40 (15.3%)
GO 3 Collaborator	19 (2.7%)	10 (3.8%)
GO 4 Leader/Manager	2 (0.3%)	0 (0%)
GO 5 Health Advocate	3 (0.4%)	15 (5.7%)
GO 6 Scholar	36 (5.1%)	25 (9.6%)
GO 7 Professional	8 (1.1%)	0 (0%)
Total	712	261

**Table 6:** Number of mappings per entrustable professional activity (EPA) chapter in total numbers as well as percentages of all EPA mappings within ETH- bachelor and USI-Master

	ETH-Bachelor	USI-Master
EPA 1	11 (2.1%)	73 (20.6%)
EPA 2	132 (24.7%)	73 (20.6%)
EPA 3	42 (7.9%)	21 (5.9%)
EPA 4	46 (8.6%)	40 (11.3%)
EPA 5	59 (11.0%)	29(8.2%)
EPA 6	195 (36.4%)	58 (16.4%)
EPA 7	48 (9.0%)	9 (2.5%)
EPA 8	0 (0%)	51 (14.4%)
EPA 9	2 (0.4%)	0 (0%)
Total	535	354

## Discussion

Curriculum mapping is generally used to assess whether the competencies of a national framework have been met, less for the evaluation of a competency-based medical curriculum itself [4, 6–8]. This study examined both, the distribution of mappings within the curriculum and the coverage of the Swiss national PROFILES framework.

### Continuity

Cardiovascular mappings were found within all six years of the medical curriculum. Cardiological content was not only present in the cardiology module of ETH-Bachelor and the circulation module of USI-Master, but also in various other modules. Analysis of distribution within each year of USI-Master revealed a lower number of different modules per year and less mappings per module compared to the ETH-Bachelor. The high number of mappings within the circulation module in the fourth year and the repetition module in the sixth year of USI-Master was to be expected. One explanation for the rather low number of mappings in the fourth and fifth year could be that the mapping of the USI-Master has gone through less iterations than the ETH-Bachelor. Some publications suggest, that the final mapping found in a curricular map is an iterative process where the individual mappings should be reviewed and a consensus between different mapping persons should be gained [4]. The sparse number of mappings in the sixth year, however, was certainly due to the lack of mapping of the clinical rotations in the USI-Master.

Our results indicate a continuity across the two institutions, which is required by an integrative curriculum with a coherent learning spiral and without unwanted redundancies and gaps [14, 31]. Taken together, hypothesis 1 has been confirmed, as the results reflect a longitudinal distribution of cardiology-related topics. This enables optimal and continuous acquisition of competencies [3, 32]. Our approach can be used for analysis of any other topic in any curriculum, which is mapped against national or international frameworks. Some topics may need a comparable continuity as the cardiovascular system; others may not need continuity in the same extend.

### External complexity

Distribution of mapping depths between ETH-Bachelor and USI-Master was significantly different. Overall, cardiovascular content within USI-Master was more complex due to the higher share of high-level mappings compared to ETH-Bachelor. This increase in complexity, which is usually discussed in the context of spiral curriculum design, is a robust and useful model for undergraduate medical education [33] and is in line with the second hypothesis which expected a higher complexity in USI-Master compared to ETH-Bachelor.

The curricular map of ETH-Bachelor was used for the creation of USI-Master, which was a novel approach in Switzerland and has resulted in a curriculum with a meaningful learning spiral concerning cardiology-related content. Thus, the creation of two consecutive study programmes in undergraduate medical education based on two curricular maps was successful in providing an integrated curriculum. However, in a recent systematic review of tools for measuring curriculum integration no study was identified, which used curriculum mapping data to measure curricular integration [34].

As stated by Harden in 2001, this study underlines the explicit notion that curricular maps are a powerful tool for managing the curriculum by making the curriculum more transparent [31]. Our evaluation method by means of two curricular maps demonstrated an increase of complexity across two institutions. An increase in complexity over time is an important part of an integrated spiral curriculum according to AMEE Guide No. 96 and should certainly also be an outcome for other organ systems.

### Internal complexity

Evaluation of mapping depths within ETH-Bachelor revealed an increasing share of level 3a mappings throughout the three years. However, level 1 and 2 mappings did not clearly decrease over time with a rather high share of level 1 mappings in the last year. This might be due to the ‘Emergency Medicine’ module in the last year, which might have comprised more basic knowledge concerning cardiovascular complaints in the emergency context compared to other modules. Overall, only year 1 was significantly different from the other two years, which might have been due to the cardiology module in the first year, where a lot of basics (on level 1 or 2) must be taught to create a basis for the following modules.

Based on these results it was not possible to fully confirm the third hypothesis that the complexity within ETH-Bachelor should increase over the three years. However, a trend towards more complex content in the later years could be identified.

Analysis of USI-Master revealed a peak of level 3 mappings (3a or 3b) within the fifth year, which decreased further resulting in the lowest share in the sixth year. This result was not in line with the third hypothesis proclaiming an increase in complexity over time in USI-Master. However, structure of USI-Master curriculum provided the explanation for the results: the cardiology module in the fourth year had a high share of 3b mappings as students perform the activities on patients in clinics. In the fifth year, mappings were predominant in the critical care module, where no patients are involved, however practical skills are performed in simulation (3a level). The repetition bloc of the cardiology content for the federal exam in the sixth year comprised no clinical days resulting in no mappings on 3b level. Level 3a mappings were also less in the repetition bloc because there was less time for this repetition bloc compared to the critical care module in the fifth year. The curricular structure can thus explain the decrease of high-level mappings within the USI-Master.

Thus, it was also not possible to fully confirm the third hypothesis that the complexity within USI-Master should increase over the three years. However, results can be explained by the curricular structure where the repetition bloc in the last year only covered the theoretical parts of the full cardiology module of the fourth year, without practical activities with patients.

### Coverage of PROFILES

The mappings for the GO chapters at both institutions were very heterogenous. There was a clear pre-dominance of the medical expert role (GO 1) at both institutions with nearly 90% of mappings at ETH-Bachelor and 65% at USI-Master. These high shares can be explained by the fact that there are nearly no PROFILES items that concern the functioning of the basic anatomy and physiology. All basic knowledge is subsumed in the medical expert chapter (GO 1.01 and GO 1.02) which resulted in very high mapping numbers compared to the other GO chapters.

The other roles beside the medical expert (GO 2 - GO 7) are frequently described as intrinsic roles [35] and their implementation in medical curricula has proven to be a difficult task [35–37]. In our analysis only the communicator role (GO 2) was frequently mapped with 15% of all GO mappings at USI-Master. The results suggested that communication with patients is not a central topic in the Bachelor, but gets more important in the Master, when students experience more clinical situation settings. Communication between patient and physician is crucial and a good implementation is therefore mandatory [38]. In the Bachelor, this result could help to check if the basics concerning communication with patients are well enough incorporated.

The following roles were very scarcely mapped at both institutions: collaborator (GO 3), leader/manager (GO 4), health advocate (GO 5) and professional (GO 7). In a study by Griewatz and colleagues the implementation of the collaborator, leader/manager and health advocate in eight medical programs in Germany revealed that the leader/manager role had the lowest curricular representation [37], which was in line with our results. The collaborator role was better covered at ETH-Bachelor and USI-Master compared to the health advocate, which was not in line with the findings of Griewatz and colleagues. However, we only evaluated the coverage of these roles in respect to cardiology and not a complete medical program like in the study by Griewatz and colleagues. Yet, the study was the most proximate study setting in literature, because we found no similar study results where only a specific part of the curriculum is assessed based on curricular data. The role of the professional was not covered by USI-Master and only scarcely at ETH-Bachelor. Yet, this role comprises important aspects which should be present in a longitudinal curriculum, like for example GO 7.2 ‘be aware of their own limits, and seek supervision when appropriate’ ([16], p.15).

The coverage of the scholar role (GO 6) was quite well at both institutions. ETH-Bachelor and USI-Master have both been created from scratch giving them the possibility to implement upcoming medical technologies and digital medicine [24, 39], which was reflected in the results.

Concerning the EPA chapters the coverage was quite similar at both institutions, yet there were some exceptions. The percentage of EPA 1 mappings (taking a medical history) was nearly tenfold higher in USI-Master than ETH-Bachelor. Taking a medical history requires some basic knowledge and principles to be successful in the clinical setting [40]. Basic knowledge and principles concerning taking a medical history are taught in the ETH-Bachelor in the anamnesis module. These mappings were not included in this analysis because the anamnesis module is not specific to cardiology but concerns taking a medical history in any discipline or setting. This result highlights the importance of the definition of the dataset for such an analysis. Furthermore, the interpretation of the results requires an in-depth knowledge of the analysed curriculum, which was present in this study.

The percentage of the following EPA chapters were roughly the same at ETH-Bachelor and USI-Master: EPA 2 (assessment of the physical and mental status), EPA 3 (prioritisation of a differential diagnosis), EPA 4 (interpretation of diagnostic tests) and EPA 5 (performance of general procedures). The results are supported by the structure of the curricula at both institutions: ETH-Bachelor comprises two modules where the finding of a differential diagnosis (EPA 3) and the interpretation of diagnostic tests (EPA 4) are included. In the practical examination course of ETH-Bachelor students learn how to examine and assess patients concerning their cardiovascular status (EPA 5). These skills are then further trained in the Master in the circulation module. They thus provide a meaningful connection between the ETH-Bachelor and USI-Master and the basis for a good transition to postgraduate training [41].

EPA 6 focuses on emergency medicine and the percentage of mappings was more than double in ETH-Bachelor compared to USI-Master. Here the results of the distribution of mappings to the modules (‘continuity’) seemed to be relevant: there were much more mappings in the emergency module at ETH-Bachelor than the critical care module at USI-Master. Upon closer inspection the data revealed that the critical care module mainly mapped SSP and nearly no EPA items. This provides an important insight for the interpretation of curriculum mapping data: mapping outcomes can vary between persons [4] therefore interpretation of results should always comprise scrutinization of original data to pay credit to possible mapping differences.

Development of a management plan (EPA 7) was more frequently mapped in ETH-Bachelor than in USI-Master. This was rather surprising, as the focus of ETH-Bachelor rather lies on the prioritization of a differential diagnosis and not the subsequent therapeutic options. Yet the basics (mapping level 1 and 2) need to be present in order to execute an EPA successfully later on [40], which was represented by the mappings in the ETH-Bachelor. Although this EPA deemed less important for students in a recent Swedish study [42], this aspect should certainly be better implemented in the USI-Master to ensure sufficient proficiency for the transition into postgraduate training.

The documentation and presentation of the patient’s clinical encounter (EPA 8) was only mapped in USI-Master and not at all in ETH-Bachelor. Documentation is, regardless of the discipline, very important [43, 44]. This topic should certainly be discussed in ETH-Bachelor already at least theoretically (level 1 and 2 mappings) to provide the basis for the execution of the clinical activity later in USI-Master (level 3a and 3b mappings).

EPA 9, the contribution to a culture of safety, was only mapped twice in ETH-Bachelor and not at all in USI-Master. This aspect seemed not to be appropriately implemented with respect to cardiology and should be better implemented in the future at both institutions.

This analysis of the coverage of the GO and EPA chapters for cardiovascular topic provides a good basis for curricular development because gaps were made visible. Overall, there were no strict rules on how many times a PROFILES item needs to be mapped or taught in a medical curriculum in Switzerland to be fulfilled to a satisfactory degree for the FLE [16, 17, 20]. Medical universities in Switzerland have had a lot of freedom in the design and execution of their medical curriculum [21]. Due to the lack of strict guidelines on the frequency of the PROFILES it was difficult to evaluate whether the number of mappings for each chapter was sufficient or not. But for the first time, a scientific analysis made the implementation of the CanMEDS roles (GO) and EPA in a medical curriculum in Switzerland visible. The results suggest that implementation of the PROFILES catalogue is not a straight-forward task [3, 20, 36, 45, 46].

### Summary, limitations and outlook

Use of curriculum mapping data across two institutions helped to visualize the continuity and the increase in complexity for cardiology over time. Furthermore, analysis of the coverage of the competencies of the Swiss national framework (PROFILES) helped to identify gaps and inequalities.

Limitations of this study are the use of the mappings, namely the number of linkages of a LO to the items of a national framework, which reflect the planned (or declared) curriculum and not the taught curriculum [31]. Number of mappings (instead of the number of LO) was used, assuming that the more PROFILES items were mapped to a LO the more complex it is. Furthermore, number of mappings per LO gives a good weighting of the LO in respect to the overall goal of the medical curriculum.

The lack of strict guidelines concerning the implementation of the national PROFILES catalogue made it difficult to evaluate whether the number of mappings over the six years were sufficient. However, as each learner needs a different exposure to knowledge and skills to develop the competency, this aspect is a general challenge of competency-based medical education [3].

In addition, continuous exposure and increase in complexity of the mapped curriculum (planned curriculum) is certainly a prerequisite for a successful learning spiral, but does not guarantee that the students do internalise it (learned curriculum) [31]. To measure the effect of a mapped curriculum on the student further analysis would have to be done in future research [47]. Furthermore it should be proven that the mapped curriculum is actually taught before evaluating the effect of the curriculum on the students [31].

Our approach can be used for any topic at any medical faculty with an integrated curriculum that is mapped against any kind of catalogue with a curriculum mapping tool (e.g. a national-wide learning outcomes). Results concerning coverage and complexity might be different for other organ systems or topics. Nevertheless, our approach shows, that (based on the curricular map) it is possible to visualize a curriculum across two universities without losing the detailed data needed for in-depth analysis to identify the possibilities for improvement.

## Supplementary Information

Supplementary Material 1: Additional data 1: Data extraction criteria for approach A and B.

Supplementary Material 2: Additional data 2: Complete study program of ETH-Bachelor and USI-Master.

## Data Availability

The datasets during and/or analyzed during the current study are available from the corresponding author on reasonable request.

## Abbreviations

CM: Curriculum Mapping
EPA: Entrustable Professional Activities
ETH: Swiss Federal Institute of Technology
FLE: Federal Licensing Exam
GO: General Objective
LE: Learning Event
LO: Learning Objective
LOOOP: Learning Opportunities, Objectives and Outcomes Platform
PROFILES: Principal Relevant Objectives and Framework for Integrative Learning and Education in Switzerland
SSP: Situations as Starting Point
USI: Università della Svizzera italiana
