# Supplementary material for: How to use curriculum mapping to ensure a coherent and coordinated learning spiral in a competency-based medical curriculum across two medical universities
Source: BMC Med Educ. 2025 Oct 7;25:1364. doi: 10.1186/s12909-025-07837-w (PMC12502588; doi:10.1186/s12909-025-07837-w)
Supplement: Supplementary file 1 — Supplementary Material 1: Additional data 1: Data extraction criteria for approach A and B. [file 12909_2025_7837_MOESM1_ESM.docx]

## Additional data 2: Complete study program of ETH-Bachelor and USI-Master

### 1^st^ Year ETH-Bachelor

|  |  | **ECTS** | | | | | | | | | | | | | | | | | | | | | | | | | | |
| --- | --- | --- | --- | --- | --- | --- | --- | --- | --- | --- | --- | --- | --- | --- | --- | --- | --- | --- | --- | --- | --- | --- | --- | --- | --- | --- | --- | --- |
|  |  | **1** | **2** | **3** | **4** | **5** | **6** | **7** | **8** | **9** | **10** | **11** | **12** | **13** | **14** | **15** | **16** | **17** | **18** | **19** | **20** | **21** | **22** | **23** | **24** | **25** | **26** | **27** |
| **Semester Week** | **1** | **Fundamentals of Medicine and the Human Body (2 ECTS)** | | | | | | | | | | | | | | | | | | | | | | | | | | |
|  | **2** | **Musculoskeletal System**  **(5 ECTS)** | | | | | | | | | | | | **Medical Interviewing Technique (2 ECTS)** | | **Molecular Genetics and Cell Biology**  **(5 ECTS)** | | | | | **Chemistry**  **(4 ECTS)** | | | | **Mathematics I**  **(4 ECTS)** | | | |
|  | **3** |  |  |  |  |  |  |  |  |  |  |  |  |  |  |  |  |  |  |  |  |  |  |  |  |  |  |  |
|  | **4** |  |  |  |  |  |  |  |  |  |  |  |  |  |  |  |  |  |  |  |  |  |  |  |  |  |  |  |
|  | **5** |  |  |  |  |  |  |  |  |  |  |  |  |  |  |  |  |  |  |  |  |  |  |  |  |  |  |  |
|  | **6** |  |  |  |  |  |  |  |  |  |  |  |  |  |  |  |  |  |  |  |  |  |  |  |  |  |  |  |
|  | **7** |  |  |  |  |  |  |  |  |  |  |  |  |  |  |  |  |  |  |  |  |  |  |  |  |  |  |  |
|  | **8** |  |  |  |  |  |  |  |  |  |  |  |  |  |  |  |  |  |  |  |  |  |  |  |  |  |  |  |
|  | **9** | **Nervous System**  **(5 ECTS)** | | | | | | | | | | | |  |  |  |  |  |  |  |  |  |  |  |  |  |  |  |
|  | **10** |  |  |  |  |  |  |  |  |  |  |  |  |  |  |  |  |  |  |  |  |  |  |  |  |  |  |  |
|  | **11** |  |  |  |  |  |  |  |  |  |  |  |  |  |  |  |  |  |  |  |  |  |  |  |  |  |  |  |
|  | **12** |  |  |  |  |  |  |  |  |  |  |  |  |  |  |  |  |  |  |  |  |  |  |  |  |  |  |  |
|  | **13** |  |  |  |  |  |  |  |  |  |  |  |  |  |  |  |  |  |  |  |  |  |  |  |  |  |  |  |
|  | **14** |  |  |  |  |  |  |  |  |  |  |  |  |  |  |  |  |  |  |  |  |  |  |  |  |  |  |  |

|  |  | **ECTS** | | | | | | | | | | | | | | | | | | | | | | | | | | | | | | | | | | | |
| --- | --- | --- | --- | --- | --- | --- | --- | --- | --- | --- | --- | --- | --- | --- | --- | --- | --- | --- | --- | --- | --- | --- | --- | --- | --- | --- | --- | --- | --- | --- | --- | --- | --- | --- | --- | --- | --- |
|  |  | **1** | **2** | **3** | **4** | **5** | **6** | **7** | **8** | **9** | **10** | **11** | **12** | **13** | **14** | **15** | **16** | **17** | **18** | **19** | **20** | **21** | **22** | **23** | **24** | **25** | **26** | **27** | **28** | **29** | **30** | **31** | **32** | **33** | **34** | **35** | **36** |
| **Semester Week** | **1** | **Cardiovascular System**  **(5 ECTS)** | | | | | | | | | | | | | | | | |  | |  | | |  | |  | |  | | | |  | | |  |  |  |
|  | **2** |  |  |  |  |  |  |  |  |  |  |  |  |  |  |  |  |  |  |  |  |  |  |  |  |  |  |  |  |  |  |  |  |  |  |  |  |
|  | **3** |  |  |  |  |  |  |  |  |  |  |  |  |  |  |  |  |  |  |  |  |  |  |  |  |  |  |  |  |  |  |  |  |  |  |  |  |
|  | **4** | **Physical Examination: Musculoskeletal and Neurological System (2 ECTS)** | | | | | | | | | | | | | | | | | | | | | | | | | | | | | |  | | |  |  |  |
|  | **5** |  | | | | | | | | | | | | | | | | | **Infection**  **(2 ECTS)** | | **Biochemistry**  **(3 ECTS)** | | | **Pathobiochemistry**  **(2 ECTS)** | | **Pharmacology**  **(2 ECTS)** | | **Mathematics II**  **(4 ECTS)** | | | | **Statistics I**  **(3 ECTS)** | | |  |  |  |
|  | **6** | **Respiratory System**  **(5 ECTS)** | | | | | | | | | | | | | | | | |  |  |  |  |  |  |  |  |  |  |  |  |  |  |  |  |  |  |  |
|  | **7** |  |  |  |  |  |  |  |  |  |  |  |  |  |  |  |  |  |  |  |  |  |  |  |  |  |  |  |  |  |  |  |  |  |  |  |  |
|  | **8** |  |  |  |  |  |  |  |  |  |  |  |  |  |  |  |  |  |  |  |  |  |  |  |  |  |  |  |  |  |  |  |  |  |  |  |  |
|  | **9** |  |  |  |  |  |  |  |  |  |  |  |  |  |  |  |  |  |  |  |  |  |  |  |  |  |  |  |  |  |  |  |  |  |  |  |  |
|  | **10** |  |  |  |  |  |  |  |  |  |  |  |  |  |  |  |  |  |  |  |  |  |  |  |  |  |  |  |  |  |  |  |  |  |  |  |  |
|  | **11** | **Kidney and Homeostasi**  **(5 ECTS)** | | | | | | | | | | | | | | | | |  |  |  |  |  |  |  |  |  |  |  |  |  |  |  |  |  |  |  |
|  | **12** |  |  |  |  |  |  |  |  |  |  |  |  |  |  |  |  |  |  |  |  |  |  |  |  |  |  |  |  |  |  |  |  |  |  |  |  |
|  | **13** |  |  |  |  |  |  |  |  |  |  |  |  |  |  |  |  |  |  |  |  |  |  |  |  |  |  |  |  |  |  |  |  |  |  |  |  |
|  | **14** |  |  |  |  |  |  |  |  |  |  |  |  |  |  |  |  |  |  |  |  |  |  |  |  |  |  |  |  |  |  |  |  |  |  |  |  |
|  | **15** | **Physiology Lab Course (3 ECTS)** | | | | | | | | | | | | | | | | | | | | | | | | | | | | | | | | | | | |

### 2^nd^ Year ETH-Bachelor

|  |  | **ECTS** | | | | | | | | | | | | | | | | | | | | | | | | | |
| --- | --- | --- | --- | --- | --- | --- | --- | --- | --- | --- | --- | --- | --- | --- | --- | --- | --- | --- | --- | --- | --- | --- | --- | --- | --- | --- | --- |
|  |  | **1** | **2** | **3** | **4** | **5** | **6** | **7** | **8** | **9** | **10** | **11** | **12** | **13** | **14** | **15** | **16** | **17** | **18** | **19** | **20** | **21** | **22** | **23** | **24** | **25** | **26** |
| **Semester Week** | **1** | **Blood, Immune System**  **(4 ECTS)** | | | | | | | | | | | | | | **Clinical Anatomy Lab**  **(5 ECTS)** | | | | | **Physics I**  **(4 ECTS)** | | | | **Statistics II (**  **3 ECTS)** | | |
|  | **2** |  |  |  |  |  |  |  |  |  |  |  |  |  |  |  |  |  |  |  |  |  |  |  |  |  |  |
|  | **3** |  |  |  |  |  |  |  |  |  |  |  |  |  |  |  |  |  |  |  |  |  |  |  |  |  |  |
|  | **4** |  |  |  |  |  |  |  |  |  |  |  |  |  |  |  |  |  |  |  |  |  |  |  |  |  |  |
|  | **5** |  |  |  |  |  |  |  |  |  |  |  |  |  |  |  |  |  |  |  |  |  |  |  |  |  |  |
|  | **6** | **Nutrition and Digestion**  **(5 ECTS)** | | | | | | | | | | | | | |  |  |  |  |  |  |  |  |  |  |  |  |
|  | **7** |  |  |  |  |  |  |  |  |  |  |  |  |  |  |  |  |  |  |  |  |  |  |  |  |  |  |
|  | **8** |  |  |  |  |  |  |  |  |  |  |  |  |  |  |  |  |  |  |  |  |  |  |  |  |  |  |
|  | **9** |  |  |  |  |  |  |  |  |  |  |  |  |  |  |  |  |  |  |  |  |  |  |  |  |  |  |
|  | **10** |  |  |  |  |  |  |  |  |  |  |  |  |  |  |  |  |  |  |  |  |  |  |  |  |  |  |
|  | **11** | **Endocrinology, Metabolism**  **(5 ECTS)** | | | | | | | | | | | | | |  |  |  |  |  |  |  |  |  |  |  |  |
|  | **12** |  |  |  |  |  |  |  |  |  |  |  |  |  |  |  |  |  |  |  |  |  |  |  |  |  |  |
|  | **13** |  |  |  |  |  |  |  |  |  |  |  |  |  |  |  |  |  |  |  |  |  |  |  |  |  |  |
|  | **14** |  |  |  |  |  |  |  |  |  |  |  |  |  |  |  |  |  |  |  |  |  |  |  |  |  |  |

|  |  | **ECTS** | | | | | | | | | | | | | | | | | | | | | | | | | | | | | | | |
| --- | --- | --- | --- | --- | --- | --- | --- | --- | --- | --- | --- | --- | --- | --- | --- | --- | --- | --- | --- | --- | --- | --- | --- | --- | --- | --- | --- | --- | --- | --- | --- | --- | --- |
|  |  | **1** | **2** | **3** | **4** | **5** | **6** | **7** | **8** | **9** | **10** | **11** | **12** | **13** | **14** | **15** | **16** | **17** | **18** | **19** | **20** | **21** | **22** | **23** | **24** | **25** | **26** | **27** | **28** | **29** | **30** | **31** | **32** |
| **Semester Week** | **1** | **Sensory Organs**  **(4 ECTS)** | | | | | | | | **From Symptoms to Diagnosis   (5 ECTS)** | | | | | **Overall Medical History (1 ECTS)** | **Physics II**  **(4 ECTS)** | | | | **Medical Imaging I**  **(3 ECTS)** | | | **Clinical Research**  **(3 ECTS)** | | | **Ethics in Medicine (2 ECTS)** | | **Precision Medicine (1 ECTS)** |  |  |  |  |  |
|  | **2** |  |  |  |  |  |  |  |  |  |  |  |  |  |  |  |  |  |  |  |  |  |  |  |  |  |  |  |  |  |  |  |  |
|  | **3** |  |  |  |  |  |  |  |  |  |  |  |  |  |  |  |  |  |  |  |  |  |  |  |  |  |  |  |  |  |  |  |  |
|  | **4** |  |  |  |  |  |  |  |  |  |  |  |  |  |  |  |  |  |  |  |  |  |  |  |  |  |  |  |  |  |  |  |  |
|  | **5** |  |  |  |  |  |  |  |  |  |  |  |  |  |  |  |  |  |  |  |  |  |  |  |  |  |  |  |  |  |  |  |  |
|  | **6** |  |  |  |  |  |  |  |  |  |  |  |  |  |  |  |  |  |  |  |  |  |  |  |  |  |  |  |  |  |  |  |  |
|  | **7** | **Skin (2 ECTS)** | | | | | | | |  |  |  |  |  |  |  |  |  |  |  |  |  |  |  |  |  |  |  |  |  |  |  |  |
|  | **8** |  |  |  |  |  |  |  |  |  |  |  |  |  |  |  |  |  |  |  |  |  |  |  |  |  |  |  |  |  |  |  |  |
|  | **9** |  |  |  |  |  |  |  |  |  |  |  |  |  |  |  |  |  |  |  |  |  |  |  |  |  |  |  |  |  |  |  |  |
|  | **10** | **Infectious Diseases (2 ECTS)** | | | | | | | |  |  |  |  |  |  |  |  |  |  |  |  |  |  |  |  |  |  |  |  |  |  |  |  |
|  | **11** |  |  |  |  |  |  |  |  |  |  |  |  |  |  |  |  |  |  |  |  |  |  |  |  |  |  |  |  |  |  |  |  |
|  | **12** |  |  |  |  |  |  |  |  |  |  |  |  |  |  |  |  |  |  |  |  |  |  |  |  |  |  |  |  |  |  |  |  |
|  | **13** |  |  |  |  |  |  |  |  |  |  |  |  |  |  |  |  |  |  |  |  |  |  |  |  |  |  |  |  |  |  |  |  |
|  | **14** | **Internal Clinical Examination** | | | | | | | | | | | | | |  | | | | **(2 ECTS)** | | | | | | | | | | |  |  |  |
|  | **15** |  |  |  |  |  |  |  |  |  |  |  |  |  |  |  |  |  |  |  |  |  |  |  |  |  |  |  |  |  |  |  |  |
|  | **16** |  |  |  |  |  |  |  |  |  |  |  |  |  |  |  |  |  |  |  |  |  |  |  |  |  |  |  |  |  |  |  |  |
|  | **17** | **Precision Medicine: Hands-on Exercises (3 ECTS)** | | | | | | | | | | | | | | | | | | | | | | | | | | | | | | | |

### 3^rd^ Year ETH-Bachelor

|  |  | **ECTS** | | | | | | | | | | | | | | | | | | | | | | | | | | | | | | | |
| --- | --- | --- | --- | --- | --- | --- | --- | --- | --- | --- | --- | --- | --- | --- | --- | --- | --- | --- | --- | --- | --- | --- | --- | --- | --- | --- | --- | --- | --- | --- | --- | --- | --- |
|  |  | **1** | **2** | **3** | **4** | **5** | **6** | **7** | **8** | **9** | **10** | **11** | **12** | **13** | **14** | **15** | **16** | **17** | **18** | **19** | **20** | **21** | **22** | **23** | **24** | **25** | **26** | **27** | **28** | **29** | **30** | **31** | **32** |
| **Semester Week** | **1** | **Emergency Medicine (2 ECTS)** | | | | | | | | | | | | | | | | | | | | | | | | | | | | | | | |
|  | **2** |  | | | | | | | | | | | **Pathology (6 ECTS)** | | | | | |  | | | | | | | | | | | | |  |  |
|  | **3** | **Oncology (2 ECTS)** | | | | | | | | | | |  |  |  |  |  |  | **Ethics & Legal Aspects & Communication**  **(4 ECTS)** | | | | **Patient Journeys**  **(3 ECTS)** | | | **Ultrasound Basics (1 ECTS)** | **Medical Technology I**  **(3 ECTS)** | | | **Foundations of Computer Science**  **(2 ECTS)** | |  |  |
|  | **4** |  |  |  |  |  |  |  |  |  |  |  |  |  |  |  |  |  |  |  |  |  |  |  |  |  |  |  |  |  |  |  |  |
|  | **5** | **Reproduction (4 ECTS)** | | | | | | | | | | |  |  |  |  |  |  |  |  |  |  |  |  |  |  |  |  |  |  |  |  |  |
|  | **6** |  |  |  |  |  |  |  |  |  |  |  |  |  |  |  |  |  |  |  |  |  |  |  |  |  |  |  |  |  |  |  |  |
|  | **7** |  |  |  |  |  |  |  |  |  |  |  |  |  |  |  |  |  |  |  |  |  |  |  |  |  |  |  |  |  |  |  |  |
|  | **8** |  |  |  |  |  |  |  |  |  |  |  |  |  |  |  |  |  |  |  |  |  |  |  |  |  |  |  |  |  |  |  |  |
|  | **9** |  |  |  |  |  |  |  |  |  |  |  |  |  |  |  |  |  |  |  |  |  |  |  |  |  |  |  |  |  |  |  |  |
|  | **10** | **Paediatrics**  **(2 ECTS)** | | | | | | | | | | |  |  |  |  |  |  |  |  |  |  |  |  |  |  |  |  |  |  |  |  |  |
|  | **11** |  |  |  |  |  |  |  |  |  |  |  |  |  |  |  |  |  |  |  |  |  |  |  |  |  |  |  |  |  |  |  |  |
|  | **12** | **Geriatrics (1 ECTS)** | | | | | | | | | | |  |  |  |  |  |  |  |  |  |  |  |  |  |  |  |  |  |  |  |  |  |
|  | **13** | **Rheumatology**  **(2 ECTS)** | | | | | | | | | | |  |  |  |  |  |  |  |  |  |  |  |  |  |  |  |  |  |  |  |  |  |
|  | **14** |  |  |  |  |  |  |  |  |  |  |  |  |  |  |  |  |  |  |  |  |  |  |  |  |  |  |  |  |  |  |  |  |

|  |  | **ECTS** | | | | | | | | | | | | | | | | | | | | | | | | | | |
| --- | --- | --- | --- | --- | --- | --- | --- | --- | --- | --- | --- | --- | --- | --- | --- | --- | --- | --- | --- | --- | --- | --- | --- | --- | --- | --- | --- | --- |
|  |  | **1** | **2** | **3** | **4** | **5** | **6** | **7** | **8** | **9** | **10** | **11** | **12** | **13** | **14** | **15** | **16** | **17** | **18** | **19** | **20** | **21** | **22** | **23** | **24** | **25** | **26** | **27** |
| **Semester Week** | **1** | **Psychiatry & Computational Psychiatry (2 ECTS)** | | | | | | | | | | | | | | | | | | | | | | | | | | |
|  | **2** | **Psychosomatic and Psychosocial Medicine (2 ECTS)** | | | | | | | | | | | | | | | | | | | | | | | | | | |
|  | **3** | **Medical Engineering II (2 ECTS)** | | | | | | | | | | | | | | | | | | | | | | | | | | |
|  | **4** | **Teamwork, Interprofessionality and Own Career (2 ECTS)** | | | | | | | | | | | | | | | | | | | | | | | | | | |
|  | **5** | **Medical Imaging II (2 ECTS)** | | | | | | | | | | | | | | | | | | | | | | | | | | |
|  | **6** | **Translational Animal Model (1 ECTS)** | | | | | | | | | | | | | | | **Emergency Refresher and Sonography**  **(1 ECTS)** | | | | | | | | | | | |
|  | **7** | **Data Science for Medicine (4 ECTS)** | | | | | | | | | | | | | | | | | | | | | | | | | | |
|  | **8** |  |  |  |  |  |  |  |  |  |  |  |  |  |  |  |  |  |  |  |  |  |  |  |  |  |  |  |
|  | **9** | **Bedside Teaching (2 ECTS)** | | | | | | | | | | | | | | | | | | | | | | | | | | |
|  | **10** | **Differential Diagnostics (2 ECTS)** | | | | | | | | | | | | | | | | | | | | | | | | | | |
|  | **11** | **377-0608-00L Translational Research Internship (7 ECTS)** (6 Weks) | | | | | | | | | | | | | | | | | | | | | | | | | | |
|  | **12** |  |  |  |  |  |  |  |  |  |  |  |  |  |  |  |  |  |  |  |  |  |  |  |  |  |  |  |
|  | **13** |  |  |  |  |  |  |  |  |  |  |  |  |  |  |  |  |  |  |  |  |  |  |  |  |  |  |  |
|  | **14** |  |  |  |  |  |  |  |  |  |  |  |  |  |  |  |  |  |  |  |  |  |  |  |  |  |  |  |
|  |  |  |  |  |  |  |  |  |  |  |  |  |  |  |  |  |  |  |  |  |  |  |  |  |  |  |  |  |
|  |  |  |  |  |  |  |  |  |  |  |  |  |  |  |  |  |  |  |  |  |  |  |  |  |  |  |  |  |

## Study program of USI-Master (year 4- year 6)

| **Study program - Master of Medicine USI** | | | | | |  |  |  |  |  |  |  |  |  |  |
| --- | --- | --- | --- | --- | --- | --- | --- | --- | --- | --- | --- | --- | --- | --- | --- |
|  |  |  |  |  |  |  |  |  |  |  |  |  |  |  |  |
| **Week** | **1** | **2** | **3** | **4** | **5** | **6** | **7** | **8** | **9** | **10** | **11** | **12** | **13** | **14** |  |
| **First semester** | | | | | | | | | | | | | | | |
| Group A | General  introduction | | Circulation | | | | Homeostasis | | | | Immune disorders | | | | Exams |
| Group B |  |  | Immune disorders | | | | Circulation | | | | Homeostasis | | | |  |
| Group C |  |  | Homeostasis | | | | Immune disorders | | | | Circulation | | | |  |
| **Second semester** | | | | | | | | | | | | | | | |
| Group A | Children and adolescents | | | | Women | | | | Bed to bedside | | Circulating cells and signaling | | | | Exams |
| Group B | Circulating cells and signaling | | | | Children and adolescents | | | |  |  | Women | | | |  |
| Group C | Women | | | | Circulating cells and signaling | | | |  |  | Children and adolescents | | | |  |
| **Third semester** | | | | | | | | | | | | | | | |
| Group A | Pediatric practice | | Critical care | | | | Trans-versal practice | Skeleton | | | Abdominal organs | | | | Exams |
| Group B | Abdominal organs | | | | Pediatric practice | | Critical care | | | | Trans-versal practice | Skeleton | | |  |
| Group C | Trans-versal practice | Skeleton | | | Abdominal organs | | | | Pediatric practice | | Critical care | | | |  |
| **Fourth semester** | | | | | | | | | | | | | | | |
| Group A | Family doctor practice | | Nervous system | | | | Head and neck organs | | | | Personality and cognition | | | | Exams |
| Group B | Personality and cognition | | | | Family doctor practice | | Nervous system | | | | Head and neck organs | | | |  |
| Group C | Head and neck organs | | | | Personality and cognition | | | | Family doctor practice | | Nervous system | | | |  |
| **Fifth semester** | | | | | | | | | | | | | | | |
|  | Elective term (6 months) | | | | | | | | | | | | | | |
| **Sixth semester** | | | | | | | | | | | | | | | |
|  | Master thesis | | | | Complementary topics, repetition blocs | | | | Dienstarztkurs (DAK) | | Complementary topics, repetition blocs | | | | Exams |
