# Supplementary material for: How to use curriculum mapping to ensure a coherent and coordinated learning spiral in a competency-based medical curriculum across two medical universities
Source: BMC Med Educ. 2025 Oct 7;25:1364. doi: 10.1186/s12909-025-07837-w (PMC12502588; doi:10.1186/s12909-025-07837-w)
Supplement: Supplementary file 2 — Supplementary Material 2: Additional data 2: Complete study program of ETH-Bachelor and USI-Master. [file 12909_2025_7837_MOESM2_ESM.docx]

## Additional data 1: Data extraction criteria for approach A and B

| **Approach A:** SSP (PROFILES) with cardiology related symptoms | |
| --- | --- |
| SSP 2.02.40 | apnoea, apnoea with arousal |
| SSP 2.02.41 | change of respiratory pattern |
| SSP 2.02.43 | chest discomfort |
| SSP 2.02.44 | chest pain |
| SSP 2.02.46 | dyspnoea |
| SSP 2.02.47 | heartburn (pyrosis) |
| SSP 2.02.50 | palpitations |
| SSP 2.02.51 | parietal thoracic pain |
| SSP 2.08.117 | vascular injuries |
| SSP 3.01.131 | abnormal blood pressure |
| SSP 3.01.133 | abnormal findings upon auscultation |
| SSP 3.01.135 | abnormal findings upon palpation |
| SSP 3.01.136 | abnormal findings upon percussion |
| SSP 3.01.137 | bradycardia, tachycardia, irregular pulse |
| SSP 3.01.147 | pallor |
| SSP 3.01.148 | pulseless patient |
| SSP 3.01.149 | transient loss of consciousness, syncope |
| SSP 3.02.150 | abnormal blood gas analysis |
| SSP 3.02.151 | abnormal cardiac enzymes |
| SSP 3.02.153 | abnormal X-rays of abdomen, chest and skeleton |
| SSP 3.02.155 | abnormal ECG |
| SSP 3.02.168 | elevated biomarkers of inflammation |
| SSP 3.02.174 | thrombopenia, thrombocytosis |
| SSP 4.04.203 | acute abdominal, epigastric pain |
| SSP 4.04.204 | acute chest; epigastric, arm, jaw, teeth pain |
| SSP 4.04.206 | acute severe dyspnea |
| SSP 4.04.214 | severe hypertension, severe hypotension |
| SSP 4.04.217 | syncope, loss of consciousness |
| SSP 4.04.218 | cardiorespiratory disturbances and arrest |
| SSP 4.05.223 | promotion of healthy life style |
| SSP 4.05.227 | shared assessment of risk and protective factors for frequent life-compromising diseases, such as cardiovascular, metabolic and oncologic diseases |

| **Approach B:** cardiology modules | |
| --- | --- |
| ETH-Bachelor | Cardiology |
| USI-Master | Circulation, Circulation repetition |
